# Time to Decide? Dynamical Analysis Predicts Partial Tip/Stalk Patterning States Arise during Angiogenesis

**DOI:** 10.1371/journal.pone.0166489

**Published:** 2016-11-15

**Authors:** Lakshmi Venkatraman, Erzsébet Ravasz Regan, Katie Bentley

**Affiliations:** 1 Centre for Vascular Biology Research, Beth Israel Deaconess Medical Center, Harvard Medical School, Boston, Massachusetts, United States of America; 2 Department of Biochemistry and Molecular Biology, The College of Wooster, Wooster, Ohio, United States of America; 3 Department of Immunology, Genetics and Pathology, University of Uppsala, Uppsala, Sweden; Universita degli Studi di Bari Aldo Moro, ITALY

## Abstract

Angiogenesis is a highly dynamic morphogenesis process; however, surprisingly little is known about the *timing* of the different molecular processes involved. Although the role of the VEGF-notch-DLL4 signaling pathway has been established as essential for tip/stalk cell competition during sprouting, the speed and dynamic properties of the underlying process at the individual cell level has not been fully elucidated. In this study, using mathematical modeling we investigate how specific, biologically meaningful, local conditions around and within an individual cell can influence their unique tip/stalk phenotype switching kinetics. To this end we constructed an ordinary differential equation model of VEGF-notch-DLL4 signaling in a system of two, coupled endothelial cells (EC). Our studies reveal that at any given point in an angiogenic vessel the time it takes a cell to decide to take on a tip or stalk phenotype may be drastically different, and this asynchrony of tip/stalk cell decisions along vessels itself acts to speed up later competitions. We unexpectedly uncover intermediate “partial” yet stable states lying between the tip and stalk cell fates, and identify that internal cellular factors, such as NAD-dependent deacetylase sirtuin-1 (Sirt1) and Lunatic fringe 1 (Lfng1), can specifically determine the length of time a cell spends in these newly identified partial tip/stalk states. Importantly, the model predicts that these partial EC states can arise during normal angiogenesis, in particular during cell rearrangement in sprouts, providing a novel two-stage mechanism for rapid adaptive behavior to the cells highly dynamic environment. Overall, this study demonstrates that different factors (both internal and external to EC) can be used to modulate the speed of tip/stalk decisions, opening up new opportunities and challenges for future biological experiments and therapeutic targeting to manipulate vascular network topology, and our basic understanding of developmental/pathological angiogenesis.

## Introduction

**Fig 1:**
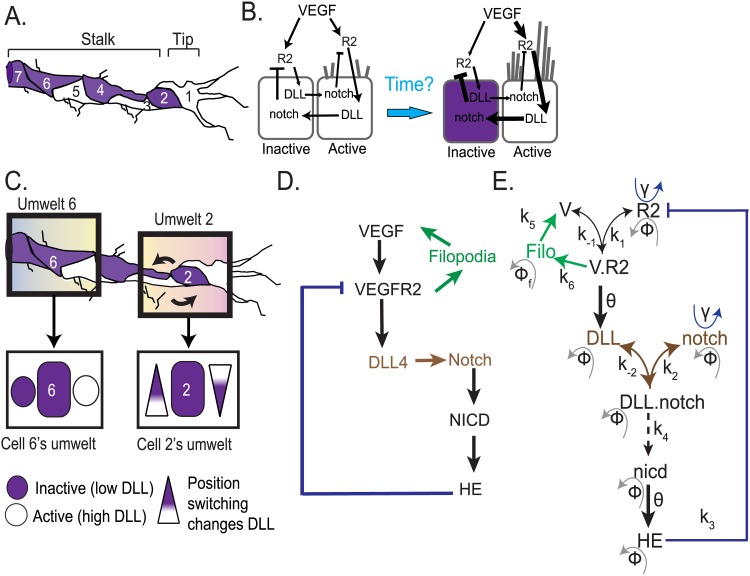
Model reaction overview. **(A)** Illustration of an angiogenic sprout, with EC in a “salt-and-pepper” pattern. White cells are the “active” cells with high DLL levels and purple cells are the “inactive” cells with high notch levels, the terms tip and stalk are positional and refer to cell 1 and cells 2–7 respectively. **(B)** Illustration of notch/VEGF lateral inhibition feedback loop between 2 adjacent ECs between quiescent state to patterned state. V.R2 = active VEGFR2 receptor, whose levels are increased in active cell1 and decreased in inactive cell2. (**C**) Representation of how different two nearby cell’s “umwelts” can be. For example, Cell 6 may have strong, previously patterned neighbors expressing near constant DLL, whereas Cell 2 is more in flux, due to position switching with cell 3 and experiences a changing level of DLL as it moves closer to different neighbors. Also each cell resides within a different region of the VEGF gradient (depicted by color gradient). (D) Brief overview of the notch-DLL interactions. Positive feedback from filopodia is shown in green, negative HE feedback in blue, lateral inhibition in brown. (**D**) Detailed 2-cell ODE model: V = VEGF ligand; R2 = VEGFR2 receptor; V.R2 = active VEGFR2; DLL = internal DLL, DLL.notch = active notch bound to DLL; nicd = NICD fragment; HE = Hes, Hey and Her combined. *γ* represents production rates while ϕ represents turnover rates (refer to Materials and Methods for details).

Angiogenesis or new blood vessel formation is essential for normal embryonic development and its dysregulation is critical to pathological processes such as wound healing and cancer [1]. Although angiogenesis is a highly dynamic morphogenesis process, with tight coordination of cellular movements leading to establishment of normal blood vessels [2,3], surprisingly little is known about the *timing* of relative molecular processes. New vessel “sprouts” budding from pre-existing blood vessels during angiogenesis are traditionally characterized at the cellular level by two phenotypes: 1) leading migratory Tip cells (Cell1, Fig 1A) that “sense” and migrate towards angiogenic environmental signals, such as vascular endothelial growth factor (VEGF) released by nearby oxygen deficient tissue and 2) following, non-migratory, Stalk cells, considered essential for creating the lumen and maintaining normal angiogenic sprouting (Cell2, Fig 1A) [4]. The selection of neighboring cells into a differential, alternating arrangement of these two states—Tip or Stalk, is hereafter referred to as endothelial cell (EC) patterning.

Using an integrated *in silico* and *in vivo* approach, we have previously contributed to the discovery that these EC phenotypes are not fixed once selected, but rather dynamically switch throughout angiogenesis, as cells rearrange their positions competing for the tip [5],[6,7]. Following a similar *in silico/in vivo* approach we also recently discovered that vascular network density is dependent on the *speed* of cellular tip/stalk selection and can be “temporally regulated” by a tissue derived factor -semaphorin3E [8]. It is therefore essential to gain a deeper understanding of the dynamical properties of the signaling network if we are to learn how specific vascular network topologies are generated and how branching could be therapeutically manipulated in disease. We present here a new detailed mathematical model and a dynamical systems study to rigorously investigate EC patterning at the single cell level and further predict whether other factors could be “temporal regulators” of EC patterning speeds.

EC patterning is known to be coordinated by notch- Delta-like Ligand 4 (DLL) driven “lateral inhibition”, where cells battle to inhibit their neighbors [9–14]. Lateral inhibition communication between the ECs can be summarized as follows (Fig 1B): VEGF receptor activation (V.R2) by VEGF ligand leads to the up-regulation of the ligand DLL, which then binds to and activates notch receptors on neighboring cells. When notch is active, the cell down-regulates V.R2. Several amplification cycles of this pathway then leads to one cell inhibiting the other’s *ability to sense*, more than the inhibition it, itself, receives. This results in EC patterning where the cell with higher DLL levels is selected as the “active” migratory cell (Tip cell) and the neighboring cell with higher notch activity takes on an “inactive” (Stalk cell) phenotype (Fig 1B). This is because higher V.R2 activation stimulates actin polymerization, filopodia and cell migration [15,16]. Most modeling studies involving EC patterning and notch-DLL signaling to date [10,17] have not focused on the specific timing of the process during angiogenic sprouting, or how different local environments of a cell in a sprout would impact this speed. Here we explicitly study and predict the effects of asynchrony in EC patterning at different regions of a sprout as well as the impact of internal regulators of Notch-DLL signaling.

The changing, local conditions around a specific EC in a real vessel constitute that cell’s unique sensory “umwelt”, its personal experience of the world. In many systems umwelt/external local environment is known to hugely impact decision-making [18,19], though its effect on cellular decisions is not often considered directly. Here, we explicitly compare simple, cellular umwelts and their impact on pattern symmetry breaking [20] and further on EC patterning speed, the “time it takes that specific cell to decide”. As examples of cellular external environment we consider local VEGF levels and external DLL representing DLL from either pre-patterned neighboring cells or artificially coated onto *in vitro* cell-culture studies [21] (Fig 1C).

Apart from the surrounding umwelt we also investigate internal cellular factors that might impact EC patterning dynamics using two likely candidates: signaling modulators- NAD-dependent deacetylase sirtuin-1 (Sirt1) and Lunatic fringe-1 (Lfng). A negative regulator the notch-DLL signaling, Sirt1 deacetylates the NICD fragment of notch, making it less stable and increasing its degradation rate [22]. In contrast, the fringe family protein –Lfng is a positive modulator of the notch-DLL signaling, as it adds N-acetyl glucosamine to notch and thereby stabilizes its interaction with DLL[23,24]. The combined effect of these internal regulators on EC patterning speed and states has never been studied before, and we hypothesize that both regulators dynamically modulate EC Tip/Stalk patterning speeds and thereby generate the abnormally hypo and hyper-branched networks observed in vivo [25–27]. Given the recent interest in the therapeutic benefits of targeting DLL and Sirt1[28–30], understanding the kinetics of the role of these regulators on notch-DLL signaling becomes essential.

Interestingly, our results indicate that the time of EC patterning i.e. determining EC states (Tip or Stalk state), is intrinsically linked to the steady state dynamics of signaling. Importantly, the model predicts the presence of partial states apart from the known Tip and Stalk states of the system. An abnormal “hybrid” state has previously been reported in a model of jagged-notch signaling [17] but was linked only to a diseased condition. Here, by considering specific cellular umwelts and internal regulation occurring in normally sprouting vessels we predict partial states are intrinsic to the normal unfolding of the process, in particular playing a role during cell rearrangement.

## Materials and Methods

### Model Construction

Reactions for the Ordinary differential equations (ODE) of the 2-cell model were written following mass-action kinetics. List of ODEs, reaction equations and parameters are provided in Tables 1, 2 and 3 respectively. Apart from parameter values obtained from [14], the 2-cell ODE model was also calibrated to match our previous experiments and simulations [5,31]. As in the agent-based model [5,32] filopodia extension and retraction are dependent on [VEGF] and they are modeled in a non-spatial manner by directly capturing the positive feedback between the VEGF ligand (V) and filopodia (filo) mediated VEGF sensing:

$$V=V*\left(1+k_{6}*filo^{n}\right)$$

where *n* represents the cooperativity in F-actin polymerization and filopodia formation (i.e. a cooperative action in n molecules of F-actin filaments leading to filopodia formation) [33]. Previous studies have shown cooperativity between at least two pathways downstream of VEGFR2 activation leading to F-actin polymerization [34].

**Table 1:** List of ODEs.

R2_cell1_′ = -*v*_1_+*v*_2_ -*v*_3_- ϕ *R2)+*γ*
R2_cell2_′ = -*v*_1_ + *v*_2_ - *v*_3_-ϕ*R2+ *γ*
V.R2_cell1_′ = *v*_1_ -*v*_2_ -ϕ*V.R2
V.R2_cell2_′ = *v*_1_—*v*_2_ -ϕ*V.R2
DLL_cell1_′ = ß+*v*_4_-*v*_5_+*v*_6_-ϕ* DLL
DLL_cell2_′ = ß+ *v*_4_-*v*_5_+*v*_6_-ϕ*DLL
notch_cell1_′ = -*v*_5_+*v*_6_ -ϕ*notch + *γ*
notch_cell2_′ = -*v*_5_+*v*_6_-ϕ*notch+ *γ*
dll.notch_cell1_′ = *v*_5_-*v*_6_-ϕ*dll.notch
dll.notch_cell2_′ = *v*_5_-*v*_6_-ϕ*dll.notch
nicd_cell1_′ = *v*_7_-ϕ*nicd
nicd_cell2_′ = *v*_7_-ϕ*nicd
HE_cell1_′ = ß+ *v*_*8*_-ϕ*HE
He_cell2_′ = ß+*v*_*8*_-ϕ*HE
filo _cell1_′ = *v*_9_- ϕ_f_*filo+ *γ*
filo _cell2_′ = *v*_9_- ϕ_f_ *filo+ *γ*

A list of the ODEs used in the 2-cell model. Each ODE represents rate of change in the protein concentration in Cell1 and Cell2. For e.g. R2_cell1_′ represents change in concentration of VEGFR2 in cell1 over time while R2_cell2_′ represents change in concentration of VEGFR2 in cell2 over time.

**Table 2:** List of reaction equations.

Reaction Equation	Reaction description
*v*_1_ = k_1_*V*R2	Forward reaction of ligand V binding receptor R2
*v*_2_ = k_-1_*V.R2	Reverse reaction of V.R2 complex dissociation.
*v*_3_ = k_3_*R2*HE^n^	Inhibition of R2 by HE.
*v*_4a_ = θ*V.R2^n^/(1+ V.R2^n^)	Gene expression of DLL by V.R2 complex.
*v*_5_ = k_2_*DLL*notch	Association of DLL and notch.
*v*_6_ = k_-2_*DLL.notch	Dissociation of DLL.notch complex.
*v*_7_ = k_4_*DLL.notch	Catalysis of the DLL.notch complex.
*v*_8_ = θ*nicd^n^/(1+nicd^n^)	Gene expression of He by nicd.
*v*_9_ = k_5_* V.R2^n^	Positive feedback between V.R2 and Filopodia.
ϕ	Degradation rate
*γ*	Production rate
n	Hill coefficient
*β*	Basal gene expression
*ϕ*_*f*_	Turnover of Filopodia.

List of reaction equations used in the 2-cell ODE model.

**Table 3:** Parameter values.

Parameter	Value	Units [reference]
k_1_	0.1	cu.sec^-1^ [14]
k_-1_	0.001	cu.sec^-1^ [14]
k_2_	0.001	cu.sec^-1^ [39]
k_-2_	0.1	cu.sec^-1^ [39]
k_3_	0.005	cu.sec^-1^ [estimated]
k_4_	0.1	sec^-1^ [14]
k_5_	0.1	sec^-1^ [estimated]
k_6_	0.001	sec^-1^ [33]
*ϕ*_*f*_	0.001	sec^-1^ [33]
θ	0.1	conc.sec^-1^ [38]
ϕ	0.001	sec^-1^ [37]
*γ*	0.005	sec^-1^ [14]
n	2	dimensionless [14,38]
*β*	0.001	sec^-1^ [estimated]

The parameters used in model construction were obtained from [14,38] [37] [33]. The parameters were also estimated to match our previous model analysis and experiments of [5,31,32,39]. Given the absence of experimental measurements of protein concentrations, we use concentration units (c.u.) as a unit to represent the relative concentrations of proteins in the model [38]. Production and degradation rates of proteins were assumed to be constant same as [14,38] [31] [40]. The initial concentrations of the proteins are set as {R2, V.R2, DLL, notch, DLL.notch, HE, filo} = {1,0,0,1,0,0,0} respectively similar to [5,39].

Gene expression of DLL and HE has been incorporated as a cooperative reaction with a hill-coefficient >1 as it has been shown previously that most transcription factors involved in gene expression (e.g. Hes, Foxc, RBP-J etc.) act through dimerization [14,35–37]. Similar gene expression equations have been used by [14] with the assumption of very fast mRNA turnover to capture the DLL expression dynamics accurately.

### Time-based analysis

Time-based model simulations were performed using ODE15s solver in MATLAB2013b (www.mathworks.com). The 2-cell ODE model (Table 1) was initialized with the parameters and initial species concentration (Table 3), and simulated over 20000 time steps, while the DLL_cell1_ and DLL_cell2_ were plotted as outputs. For calculating the time taken to pattern, the DLL_cell1_ and DLL_cell2_ values of the 2-cells were measured and the time taken to diverge between the active state with high DLL (defined as DLL >13 c.u. as determined by more than 70% of VEGF combinations) and the inactive state with low DLL (defined as DLL < 0.5 c.u. seen when VEGF combinations are negligible to allow pattering) was calculated and plotted.

### Changing DLL_ext_ values

**Fig 2:**
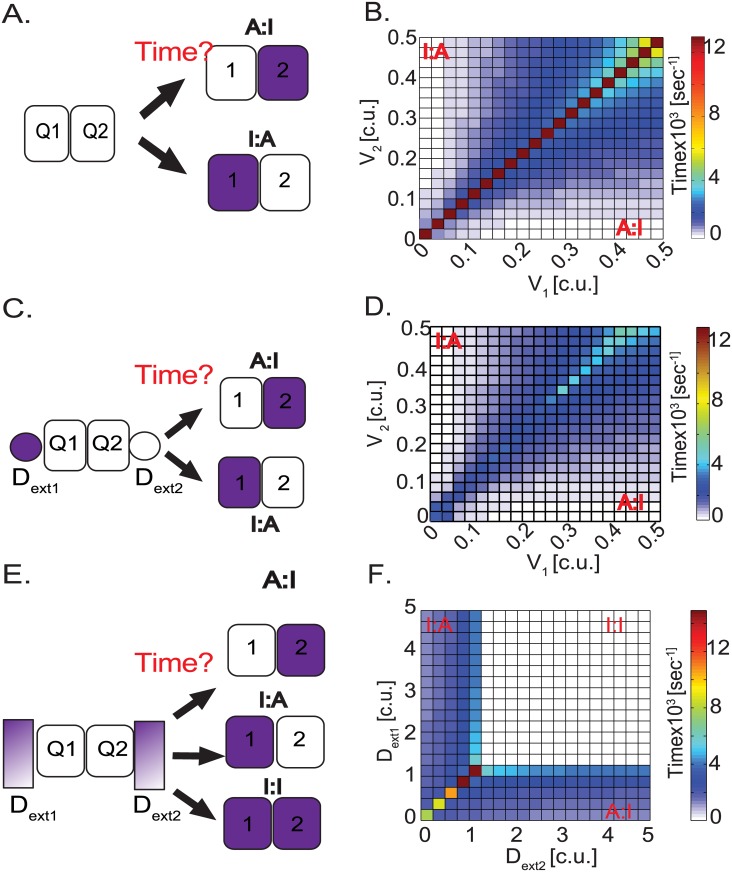
Effect of local umwelt on EC patterning speeds. (**A**) Illustration of simulation where the 2-cell model is initialized as 2 quiescent ECs (Q1 and Q2) in an environment of VEGF gradient and we ask how long does it take the 2-cell model to pattern into A:I (Active cell1:Inactive cell2) or I:A (Inactive cell1:Actitve cell2). (**B)** Matrix plot of the 2-cell system patterning speeds under different combinations of V_1_ and V_2_. **(C)** Illustration of simulation where 2-quiescent cells (Q1 and Q2) bounded by neighbors with high DLL (D_ext1_) and low internal DLL (D_ext2_) respectively. (**B**) Matrix plotting the patterning speeds under different combinations of V_1/2_ and constant D_ext_. **(C)** Illustration of simulation where 2-quiescent cells (Q1 and Q2) bounded by neighbors that can have different DLL_ext1/ext2_ values at start of each simulation. (**B**) Matrix plotting the patterning speeds under different combinations of D_ext1/ext2_. In all conditions Active cells with high DLL are white, inactive cells with high notch are purple. Patterning time is indicated as a color scale where red indicates slow patterning speeds and color ranges of blue to white indicate faster patterning speeds.

To capture the effect of changing environmental DLL, we introduce D_ext1_ & D_ext2_ in Fig 2 that represent the external DLL4 present in the environment. For simulating positional change each cell ‘sees’ an increase/decrease in the D_ext_ given by the following equations [32]:

$$DLL_{cell1}=DLL_{cell1}+DLL_{ext2}$$

$$DLL_{cell2}=DLL_{cell2}-DLL_{ext1}$$

### Sirt1-Lfgn1 model

Sirt1 deacetylates the NICD fragment of Notch, making it less stable and increasing its degradation rate[22] and this effect was captured by inclusion of Sirt1 to the degradation parameter associated with NICD (γ_nicd_ = γ_nicd_*[nicd]*[sirt1]). Lfng1 stabilizes interaction with Notch- DLL4[23,24] interaction and was modeled as the k_2lfgn_ = k_2_ * DLL_cell1/cell2_ * notch * lfng1, where k_2_ is the affinity parameter between [DLL_cell1/cell2_] and [notch].

### Steady State analysis

All steady state analysis of the 2-cell ODE model was carried out using the AUTO bifurcation toolbox in XPPAUT (http://www.math.pitt.edu/~bard/xpp/xpp.html). The 2-cell model was initialized with parameters and initial concentrations shown in Tables 1 and 3 respectively to start with a pre-patterned condition of cell1 = active and cell2 = inactive.

### Changing Sirt1 and Lfng

We analyze the effect of simultaneously perturbing Sirt1 and Lfng under a constant D_ext1_ = 1 c.u. and D_ext2_ = 2 c.u.. Cell1 switches its state from A↔I with decreasing Sirt1 levels (i.e. Sirt1 <1 S3A Fig), but with increasing Sirt1 (i.e. Sirt1>1) it undergoes an A↔pA switch. The Sirt1 threshold concentration needed for cell1 to switch from A↔I is significantly decreased with increasing Lfng values (indicating the tug of war between the pro-and anti- Notch modulators). Cell2, which starts out as an inactive cell undergoes an I↔pI switch at low values of Sirt1 and a pI↔A switch at higher values of Sirt1. At higher concentrations of Lfng, the threshold concentrations of Sirt1 needed to make the pI↔A switch are increased, indicating the strong tendency of the inactive cell2 to remain in pI state at very high Lfng values (S3B Fig).

### Clustering

Hierarchical clustering of species concentration from all the bifurcation diagrams were performed using Cluster software [41,42] and the output was drawn using Java TreeView software[43].

### Robustness/sensitivity analysis

To test the sensitivity of the parameters used to predict bistable behavior we conducted a robustness analysis[40,44]. Bistability was calculated as shown previously [40] using randomly generated parameter sets and VEGF as the bifurcation parameter. A parameter space of 200 new parameter sets was randomly generated using Latin Hypercube sampling [45,46]. Each parameter in the set had a variation of ±10% to ±50% from its nominal values and was tested for presence of bistable outcome. S4 Fig shows the percent bistability, i.e., the proportion of parameter sets that are capable of bistability when all parameters are varied. There is only a 40% decrease in percent bistability when parameters are varied ±50% of the nominal values, indicating that overall the parameter sets are robust to generate bistable outcomes.

## Results

### Constructing the mathematical model

In order to analyze the core dynamics of EC patterning, we first developed a detailed ODE model of VEGF-notch-DLL signaling. Throughout this paper when discussing EC patterning dynamics Tip cells are referred to as “Active” cells (with high DLL4 signaling) while the Stalk cell are referred to as “Inactive” cells (with high notch signaling). An outline of the signaling interactions included in our ODE model is shown in Fig 1D, while a more detailed reaction map the ODE model, including its reaction parameters is shown in Fig 1E. Specifically, VEGF ligand (V) reversibly binds the VEGFR2 receptor (R2, we are primarily interested here in the activity of VEGFR2 as the VEGFR1 receptor acts as a decoy or sink, binding VEGF but giving weak signal [47]), forming the active receptor complex (V.R2). The receptor complex induces DLL ligand gene expression (DLL), which then reversibly activates the notch receptor (notch) of the *neighboring* cell. Activated notch-DLL complex (DLL.notch) is irreversibly catalyzed to form an NICD fragment (nicd), which, in turn, induces transcription of the HE set of gene repressors. For the sake of simplicity we lump all known NICD-induced repressors, namely the HES, HEY and HER family proteins, into a single species, HE [48–50]. Through a negative feedback mechanism, HE represses the activity of R2[50], thus leading to a decrease in the number of polymerized filopodia and lamella protrusions. To capture this impact on filopodia and other cell shape changes in our non-spatial model we considered the following: an increase/decrease in filopodia leads respectively to increased/decreased perception of VEGF (V) by the cell, capturing the active perception properties of filopodia recently discussed in [32] (refer to Materials and Methods for details on model equations and parameters). To model two adjacent EC cells, the above signaling network is reproduced in each cell, with DLL of one cell activating notch in its neighbor. The model from hereon would be referred to as the 2-cell model. The parameters for model building were obtained from previous VEGF-notch-DLL models [5,14,32]. The three types of external and internal modulators to the 2-cell model were incorporated as follows: 1) V_1_ and V_2_ as ligand values that modulate the localized VEGF seen by each cell in the 2-cell model, 2) D_ext1_ and D_ext2_ as species that represent the external DLL from cells neighboring the 2-cells in our model (to differentiate between the internal DLL and external DLL species we use DLL for the former and D_ext_ for the latter) 3) Sirt1 and Lfng as species that modulate nicd degradation and DLL.notch binding affinity, respectively, in both cells of the 2-cell model.

### Relative VEGF levels perceived by the cells dramatically impact patterning speed

We first simulated a simple scenario varying the two cells relative experiences of VEGF and then build to more complex scenarios where the umwelt contains cells that have made their decisions earlier, and are either stationary or in the process of switching positions. We analyzed the time required for two un-patterned (quiescent) ECs to establish an active/inactive pattern, where one cell is active (A) and the other inactive (I), upon exposure to different levels of the external modulator VEGF. To this end, we simulated the 2-cell model starting from a quiescent condition (see Materials and Methods for initial conditions), exposed the two cells to different VEGF levels, and asked how long it takes them to “pattern out” into Inactive:Active (I:A) or Active:Inactive (A:I) states for cell1:cell2, respectively (Fig 2A). This condition represents an *in vivo* scenario of ECs in a quiescent vessel experiencing VEGF to initialize the tip cell required to lead a new sprout. The time taken to pattern out under each set of initial concentrations of VEGF was plotted as a time matrix plot (Fig 2B). Conditions where V_1_ (VEGF seen by cell1) > V_2_ (VEGF seen by cell1) pattern out into a scenario where cell1 is active and cell2 is inactive (A:I), while conditions where V_1_ < V_2_ pattern into a scenario where cell1 is inactive and cell2 is active (I:A). When the VEGF “seen” by both the cells is similar (i.e. when V_1_ = V_2_ = 0, the diagonal in Fig 2B), the 2-cell model takes a much longer time to pattern out. Increasing the difference between the V each cell sees (akin to a steeper VEGF gradient) decreases the time needed for the two cells to pattern. Thus the model predicts that the local levels of V to each cell can modulate the timing of *collective* selection of active/inactive states among neighbors. This result overall matches previous results seen with a single change between a VEGF gradient or uniform VEGF distribution using an agent based model of VEGF/notch signaling [31], and is in line with the many theoretical studies showing the impact of increasing inhomogeneity in the system to advance symmetry breaking and pattern formation [20].

### Pre-patterned neighboring cells speed up later tip/stalk decisions

Apart from the influence of environmental VEGF changes on EC patterning, notch-DLL mediated interaction is strongly influenced by the states of other neighboring cells around cell1 and cell2 within the multicellular sprout. For example, an inactive neighbor next to quiescent cell1 and active neighbor next to quiescent cell2 would influence the time of patterning in the 2-cell model under the influence of external VEGF (Fig 2C). To model the effect of these neighbors in our 2-cell system, we simulate a bolus of external DLL (D_ext_) that mimics the local DLL neighborhood of cells adjacent to the 2-cells, external to the 2-cell model itself. D_ext_ bolus’s are added, bracketing the 2-cell model with cell1 experiencing no lateral inhibition from its neighbor (D_ext1_ = 0 c.u.) and cell2 experiencing strong inhibition (D_ext2_ = 1 c.u.) from its neighbor (white cells have high DLL activity and purple cells have high notch activity, Fig 2C) We re-simulate the 2-cell model with D_ext_ added under the set of different V conditions previously simulated (Fig 2B) and ask how long does it now take the 2-cells to pattern into active and inactive states. We find the patterning speeds seen in the presence of D_ext_ (Fig 2D) are significantly faster than in the absence of pre-patterning D_ext_ (Fig 2B). This result indicates that presence of ΔD_ext_ (i.e. D_ext2_- D_ext1_) > 0 units between the cells (in the form of neighboring cells that are already differentially patterned) can significantly influence the speed of patterning of cells whose states are not yet selected, even when the two cells see almost equal V levels.

Next we simulated various levels of D_ext_ bolus levels seen by the 2-cell model representing an *in vivo* scenario of 2-cells experiencing neighbors which are themselves transitioning through different stages of notch-DLL signaling (Fig 2E). In conditions when the 2-cell model was initialized with D_ext1_ > D_ext2_ the 2-cells patterns out quickly into an I:A pattern. Alternatively, when initialized with D_ext1_ <D_ext2_ the inverse A:I pattern is reached. Finally, very high D_ext1_ and D_ext2_ values lead to the I:I state (Fig 2F). The corresponding patterned states are shown in S1 Fig. These results indicate that the patterning dynamics is highly influenced by changing D_ext_ dynamics in neighbors. Therefore, the time required for individual cell states to establish could vary dramatically at different regions in a sprout, dictated by the current state of the surrounding cells. In contrast to the above simulation, which was performed with a constant bolus of D_ext,_ we next study the effect of continuously modulating D_ext_ values by cell movement (as seen in an actively growing angiogenic sprout) on both the patterning speed and modulate pattern switching.

### Position-switching modulates the rate of state-switching

**Fig 3:**
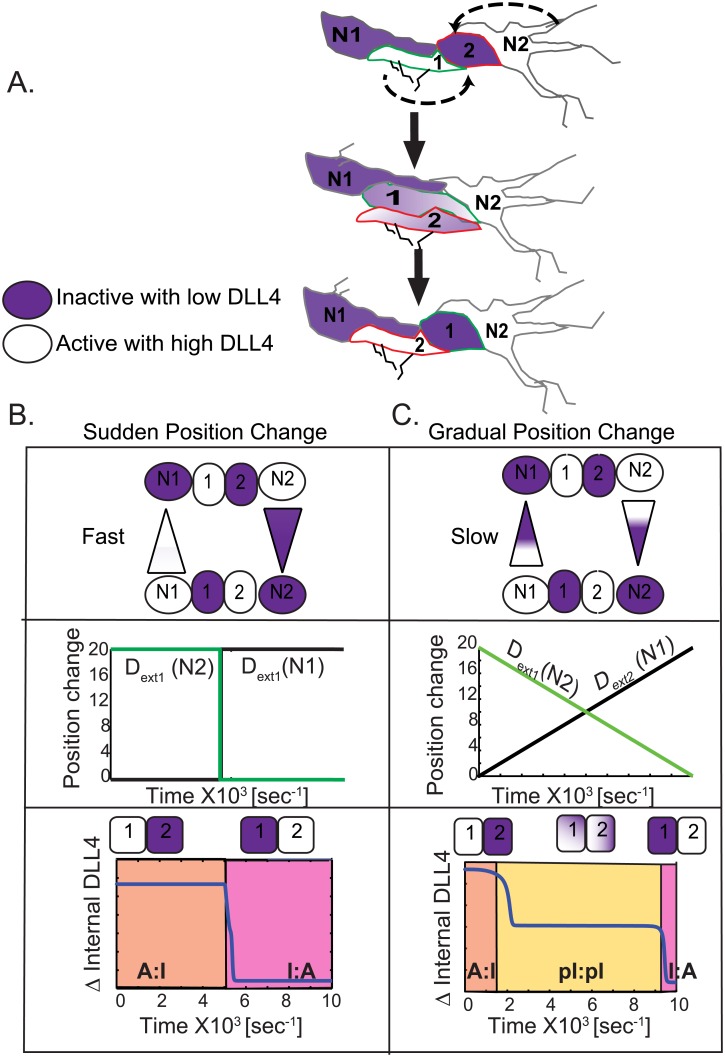
Position switching modulates the rate of state switching. (A) In vivo scenario of 2 pre-patterned cells (cell1 and cell2, A:I) in a sprout bounded by neighbors N1 (with low DLL) and N2 (with high DLL) changing positions and switching states to I:A direction showed by dashed black arrows. There is an intermediate sate where cell1 and cell2 lie next to each other. **(B)** In silico illustration and measurement of state change over time with a sudden movement of cell1 away from N1 (seeing decreasing DLL) and cell2 moving toward N2 (seeing increasing DLL). (**C**) In silico illustration and measurement of state change over time with a gradual movement of cell1 away from N1 (seeing decreasing DLL) and cell2 moving toward N2 (seeing increasing DLL).

Once established, the differential active/inactive pattern is not static instead it is constantly disrupted and re-established as cells move and interchange positions during cell rearrangement [7]. Previously, simulations predicted that the change in connectivity of neighbors during fusion of EC (anastomosis) could induce “phenotype flipping” of active cells to an inactive state and vice versa, due to the new frontier created with a high DLL neighbor during fusion [5]. However, no previous study has considered the precise influence of dynamically moving cells, shuffling along the vessel, in determining the speed and mechanistic details of state switching. We therefore next asked the question “how do continuous changes in the positions of the cells relative to their neighbors impact on the time it takes for an established differential pattern to switch to the opposite pattern?” To answer this, we considered a simple physiological scenario where an active cell1 moves to overtake its inactive cell2 neighbor such that their junctions with bracketing neighbors N1 (inhibited) and N2 (active) change over time (illustrated in Fig 3A). To capture this behavior in the non-spatial ODE 2-cell model, we vary the external DLL levels such that cell1 sees a decrease in its neighboring D_ext1_ as it moves away from N1 towards N2 (Fig 3A), and cell2 sees an increase in its neighboring D_ext2_ as it moves from N2 towards N1. For simplicity, we consider two possible cell rearrangement speeds of the two-cell system—(a) An abrupt/sudden position switch, such that the cells in the 2-cell model experience a dramatic switch in the D_ext_ levels from N1 and N2 (Fig 3B) or (b) a more gradual overtaking scenario where the 2-cell model experiences gradually increasing/decreasing values of D_ext1_(N1) and D_ext2_(N2) (Fig 3C). In light of observations of cell-movement timescales on the order of hours in growing sprouts [7], the second scenario of gradual position changing can be considered more prevalent *in vitro/in vivo*, though more rapid changes could also potentially occur.

The 2-cell model indeed undergoes a rapid symmetrical A:I to I:A transition when the cells abruptly switch position relative to N1 and N2 (Fig 3B). In the second scenario, using gradual position change, the simulation yielded very interesting dynamics (Fig 3C). The 2-cell model no longer undergoes a symmetrical A:I to I:A switch. Contrary to the complete, rapid switch seen in Fig 3B, in this scenario both the cells of the 2-cell model stabilize at an intermediate level of internal DLL for a long period of time (Fig 3C, yellow region). During this intermediate level the internal DLL concentrations of the 2-cells are not as high as the active cell1 nor as low as the inactive cell2, thus we term this a “partial inactive” (pI) state. It is interesting to note that pI state occurs for the duration when the N1 and the N2 cells are exerting equal or close to equal lateral inhibition (i.e. D_ext1_ ≅ D_ext2_) on the 2-cell model. Importantly, the pI state is not transient, and is maintained as long as the 2-cell model is not able to get clear polarized signal from the neighbors (i.e. in this case lateral inhibition values that are differential by at least 10%). The pI state further indicates the complexity that cell position switches add to this system, changing notch-DLL signaling from generating the expected binary dynamics to one with a third, intermediate stable state. The concept of “salt-and-pepper” patterning is therefore not necessarily absolute and not always binary, as previously thought. The extent of lateral inhibition from neighboring cells when positions are changing not only gives rise to the presence of intermediate patterning states, but also determines the time ECs spend in deciding to move away from this intermediate state, creating a potentially highly variable temporal landscape for decision making along the sprout.

### Position changes induce multi-step, switch-like transitions in EC patterning

**Fig 4:**
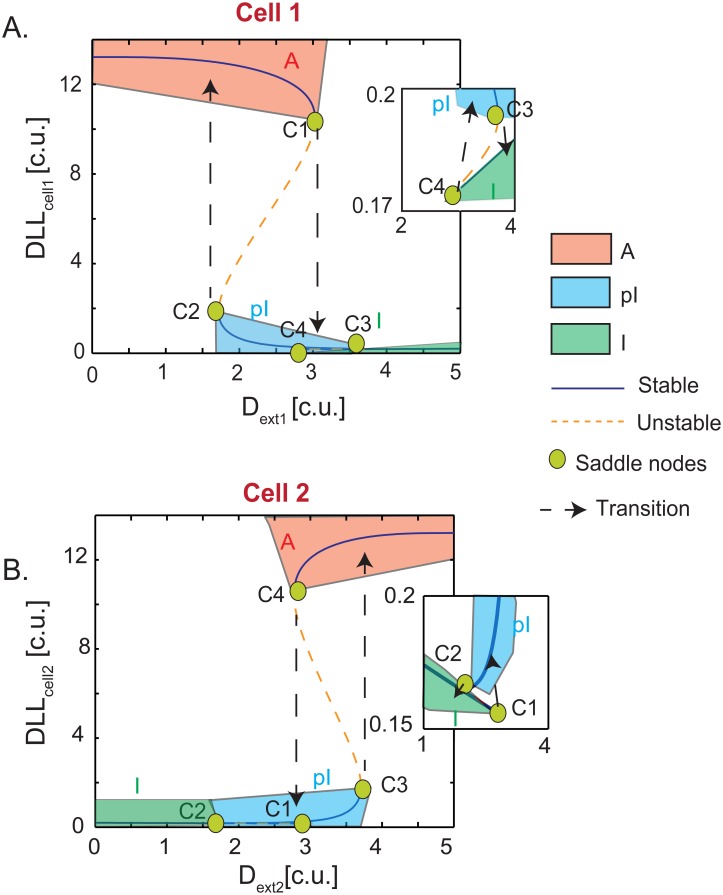
Multistep EC pattern switching modulated by position changes. (**A**) Bifurcation diagram of cel1 that starts as an active cell with high internal DLL (DLL_cell1_) and with changing DLL_ext_ values converts to an inactive cell with low DLL. (**B**) Bifurcation diagram of cell2 that starts as an inactive cell with low internal DLL (DLL_cell2_) and with changing DLL_ext_ converts to an active cell. Blue solid lines indicate a stable steady state while dashed green lines indicate unstable steady states. Orange regions = Active state, blue regions = pI states, green regions = Inactive states. The saddle nodes are marked as green circles. The dashed black arrows indicate the direction of state switching with changing DLL_ext1/ext2_ values.

To analyze the time lag seen in pattern switching under certain conditions and the emergence of a partial state during gradual cell rearrangements, we next performed bifurcation analysis on the steady state behavior of the system (Fig 4). Simulations were initialized with the 2-cell model in a pre-patterned A:I state and the D_ext_ values were increased with cell1 seeing more D_ext1_ (Fig 4A) while cell2 saw decreasing D_ext2_ (Fig 4B, refer to Materials and Methods for details). With changing of the D_ext_ values, the internal DLL_cell1_ value starts to decrease as cell1 encounters higher D_ext1_ and internal DLL_cell2_ value starts to increase as cell2 experiences lower D_ext2_. At a threshold of D_ext1_, cell1 experiences a high degree of lateral inhibition that switches it from the completely active state (C1 saddle node in Fig 4A) to a “partial Inactive” (pI) state (C2 saddle node in Fig 4A). This abrupt transition in cell1 leads to a weakened lateral inhibition and a slight increase in DLL_cell2_ (C1 to C2 saddle node transition in Fig 4B). DLL_cell2_ values in this state are not comparable to that of an active cell; rather, they fall within the same range as those of the “partial Inactive” (pI) cell1 (Fig 4B and zoomed inset). Further increase in D_ext1_ pushes cell1 past another threshold, where it loses its pI state and becomes a fully inactive cell (C3 to C4 saddle node transition in Fig 4A and zoomed inset). At the same time, pI state of cell2 loses all lateral inhibition and become completely active (C3 to C4 saddle node transition in Fig 4B).

These simulations intriguingly predict that cells must undergo two bistable switches to change from a completely active to completely inactive state while dynamically shuffling through an angiogenic sprout. I.e. cell1 can only be moved from being A↔pI↔I and cell2 from being I↔pI↔A. The reversible arrows used at each transition step indicate the flexible bistable nature of the system to return back to its previous state rather than continue if the D_ext_ conditions do not persist. It is to be noted that the switches are also asymmetrical, i.e. when cell1 makes a big transition from A↔pI, cell2 makes a smaller transition from I↔pI (and not as would be expected a symmetrical I↔A switch). Continued shuffling of cells on the angiogenic sprout will move both cells away from the pI position i.e. pI↔I (cell1) and pI↔A (cell2) thus finishing a complete switch in A↔I patterning. Biologically, a two-stage bistable switch around the pI:pI state would create a very efficient threshold/flexibility in pattern switching. If the neighboring cells D_ext_ values are not high/low enough to move beyond the pI state, the cells would be able to return back to their original states rapidly.

### Sirt1 and Lfng modulate EC patterning speeds

**Fig 5:**
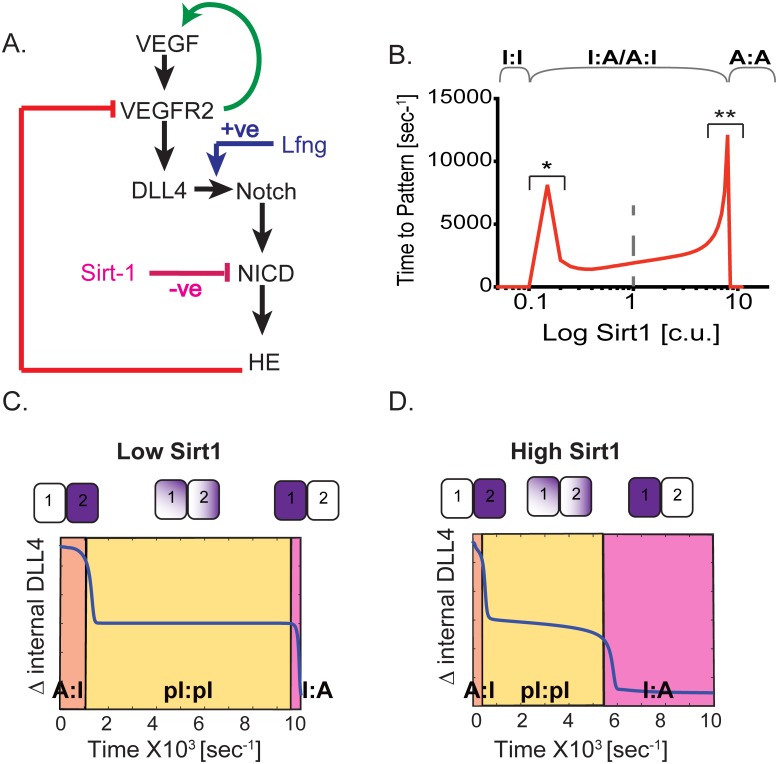
Effect of Sirt1 on EC patterning speeds. **(A)** Overview of the 2-cell model interaction with a positive regulation by Sirt1 (pink) and a negative regulation by Lfng (blue). **(B)** EC patterning speeds measured with different Sirt1 concentrations. Sirt1 = 1 c.u. optimal model, * and ** represent the threshold Sirt1 levels to transit from I:I →I:A/A:I and I:A/A:I→ A:A states respectively. State change in internal DLL values measured over time at **(C)** decreased Sirt1 (Sirt1 = 0.5 c.u.) and **(D)** increased Sirt1 (Sirt1 = 2 c.u.).

Apart from external umwelt conditions discussed above, we hypothesized that EC patterning speed can also be modulated by internal signaling modulators of the notch-DLL pathway. As a first step we focus on NAD-dependent deacetylase sirtuin-1 (Sirt1) and Lunatic Fringe-1 (Lfng) as they provide contrasting regulation of the notch system. Sirt1 deacetylates the NICD fragment of notch, making it less stable and increasing its degradation [22,26], thus acting as a negative regulator of the notch-DLL signaling. Using agent-based modeling, we previously observed that Sirt1 knock-out models may slow down EC patterning, though we did not investigate the full dynamical properties within that model[22]. Lfng1 (Lfng) acts as a positive regulator of the notch-DLL signaling by stabilizing and strengthening the notch-DLL interaction (Fig 5A).

The effect of Sirt1 on notch signaling and EC patterning was modeled by adding a “Sirt1” input species to the 2-cell model. Sirt1 increases the degradation parameter of NICD (the active fragment of notch; [Sirt1] = 1 corresponds to our original model, refer to Materials and Methods for details). Low levels of Sirt1 push the 2-cell model into an I:I state while very high values of Sirt1 push it into an A:A state (Fig 5B). These results are in line with *in vivo* and *in vitro* experimental data showing the inhibition of EC sprouting with Sirt1 loss-of-function, and hypersprouting with Sirt1 gain-of-function [25,27]. We also notice that not only does high/low Sirt1 prevent patterning, but close to the threshold values (0.15 c.u. (*), 8.5 c.u. (**) shown in Fig 5B) the patterning speeds are significantly increased, predicting that changes in Sirt1 concentration alone could have a dramatic effect on EC patterning speed.

We next asked, how does Sirt1 affect the speed of state switching in situations where the cells are also likely to be in the process of switching positions, and experiencing changing levels of D_ext_ from their neighbors. We simulated the 2-cell model with initial conditions as shown in Fig 3C with different values of Sirt1. Compared to the condition with Sirt1 = 1 (control wild type condition) simulated previously (Fig 3C), decreasing Sirt1 (Sirt1 = 0.5 c.u.) delays patterning (Fig 5C), while increasing Sirt1 levels (Sirt1 = 2 c.u.) allows for faster state switching (Fig 5D). Importantly, significant change in the partial states in low or high Sirt1 conditions were observed, in that lowering/increasing Sirt1 levels increased/decreased the time spent in partial states respectively (Fig 5C and 5D). At lower Sirt1 levels there is more NICD accumulation in the system, as it is not being degraded as fast. This leads to an increased lateral inhibition and a scenario where the 2-cells fight more strongly with each other for a longer time before deciding to switch patterns. Physiologically this simple model explains a complex *in vivo* scenario where cells in the angiogenic sprout are rearranging under different Sirt1 gene expression conditions, revealing that the precise Sirt1 level at a given time will impact greatly on state switching speed given dynamic cell rearrangement.

In contrast to Sirt1, which destabilizes the notch-DLL signaling, Lfng stabilizes it by increasing affinity of DLL to notch[24]. This contrasting role of Lfng is evident when increased (Lfng = 2 c.u.) values of Lfng compared to control (Lfng = 1 c.u.) cause an I:I state while decreased values of Lfng (Lfng = 0.5 c.u.) cause an A:A state (S2A Fig). These results are in line with previous *in vitro* and *in vivo* experiments, that have shown knockout of fringe causes hypersprouting angiogenesis [51] while overexpression causes increased HE expression in developing brain cells [52]. The effect of Lfng on position induced state switching is also contrasting to Sirt1 with low Lfng (Lfng = 0.5 c.u.) decreasing the time pattern switching takes (S2B Fig) compared to a condition of high Lfng expression (Lfng = 2 c.u.), which increases the time it takes (S2C Fig).

Taken together, these results indicate a modulatory influence of Sirt1 and Lfng on EC patterning speed, and more importantly, their regulation of the patterning dynamics (both time and state) in the angiogenic sprout where cells are constantly rearranging and thus experiencing varied lateral inhibition from neighbors.

### Sirt1 modulates partial state dynamics

**Fig 6:**
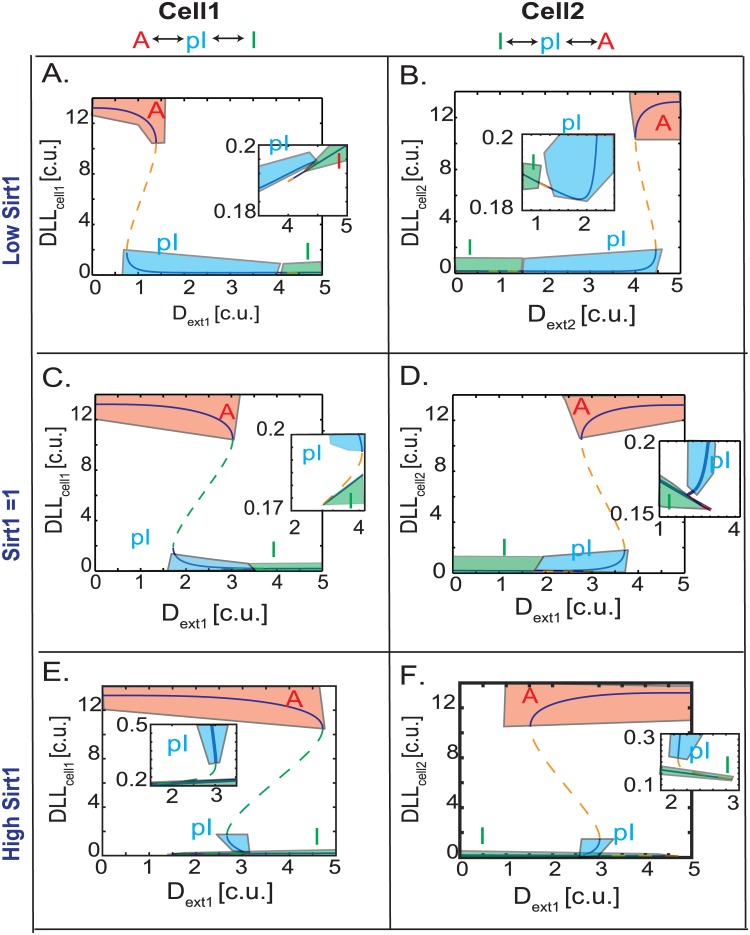
Sirt1/Lfng together modulate EC patterning dynamics. Bifurcation diagrams of internal DLL steady state levels in the 2-cell model perturbing DLL_ext_ levels continuously and changing Sirt1 values of (A,B) Low Sirt1 = 0.5 c.u., (C,D) control/wild type Sirt1 = 1 c.u. and (E,F) high Sirt1 = 2 c.u. in cell1 and cell2. Blue solid lines indicate a stable steady state while dashed green lines indicate unstable steady states. Orange regions = Active state, blue regions = pI states, green regions = Inactive states.

We next analyzed the steady state behavior of the model with changing Sirt1 values. The simulations were initialized with control/wild type condition i.e. the 2-cell model experiencing changing levels of D_ext in_ the presence of basal Sirt1 = Lfng = 1 c.u. (Fig 6C and 6D) and then compared to simulations where Sirt1 values were increased or decreased from the control. In a condition with lower Sirt1 (Sirt1 = 0.5 c.u.) the range of D_ext differences_ that lead to partial Inactive state (pI, blue region Fig 6A and 6B) of the 2-cell model is increased compared to the control condition of Sirt1 = 1. In contrast, with increased Sirt1 (Sirt1 = 2 c.u.) the range of DLL_ext_ differences that lead to pI state (blue region in Fig 6E and 6F) is reduced compare to control. Decreasing Sirt1 values increases notch signaling; allowing for more lateral inhibition and a prolonged pI:pI state in the A:I ↔ pI:pI↔ I:A transition of the 2-cell model. Increasing Sirt1 values decrease lateral inhibition and aid patterning of the 2-cell system, allowing it to break away from the pI:pI state. Further steady states characterizations of partial state altering both Sirt1 and Lfng1 (the positive regulator of notch-DLL signaling) revealed the presence of an additional partial state—partial active (pA) (S3 Fig).

### Characterization of the partial state

**Fig 7:**
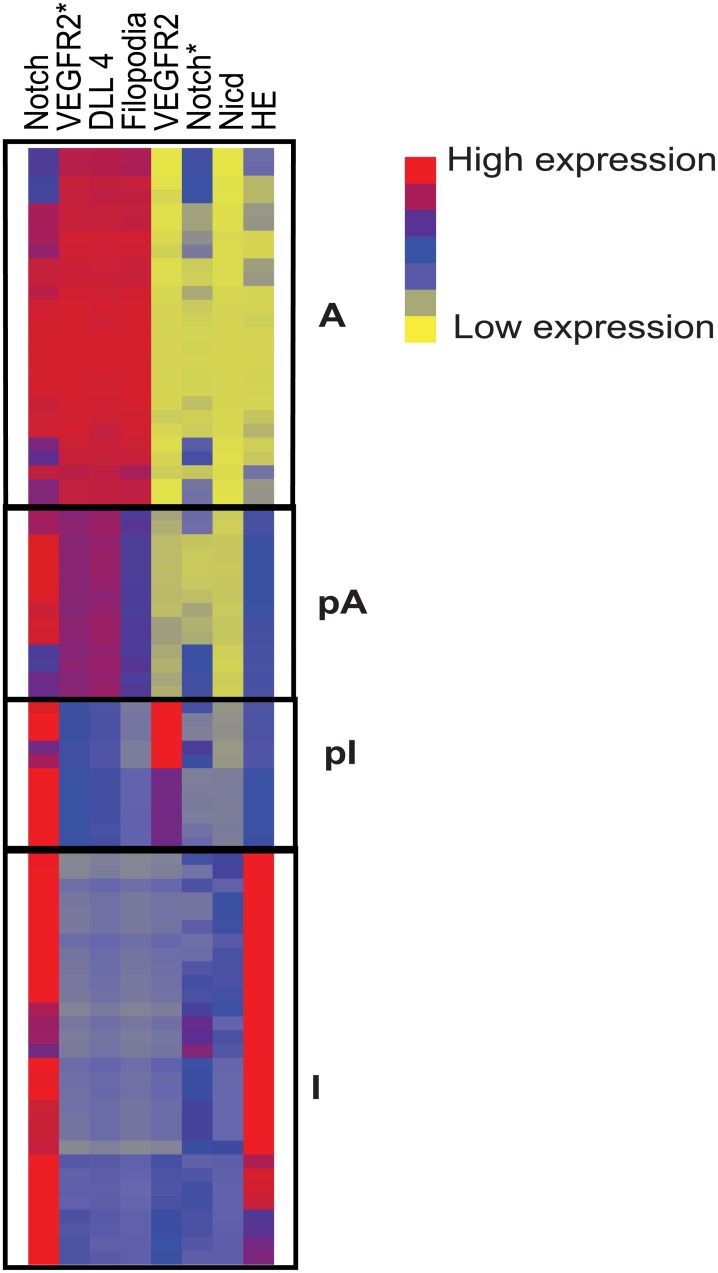
Characterization of the partial states across species of the model. Hierarchical clustering of all the species of the 2-cell model VEGFR2- Total inactive receptor; VEGFR2*- Active receptor, notch- Total inactive notch. notch* = Active notch receptor. While the columns represent the proteins, each row represents protein levels in individual cell under different simulation conditions.

Our bifurcation studies so far have focused only on internal DLL levels as an output to observe state switches. To fully characterize the mathematically distinct partial states we discovered across simulations and compare them to the fully active and inactive states we analyzed the expression/activity of all internal molecular species of the 2-cell ODE model in representative stable states from every distinct region on the bifurcation diagrams shown in Figs 4 and 6 and S3 Fig. For example, we picked A:I state at D_ext1/ext2_ = 0.007 c.u. as well as a pI:pI state at D_ext1/ext2_ = 3 c.u. and an I:A state at D_ext_ = 4.5 c.u. (Fig 4A and 4B). The results were then organized using hierarchical clustering (Fig 7). Columns represent the different protein species in the model, while individual rows represent protein levels in each cell. Interestingly, the stable cell states observed across all types of environmental perturbations cluster into four distinct branches: fully active (A), partial active (pA), fully inactive (I) and partial inactive (pI) (Fig 7). The partially active (pA) cells exhibit more notch-driven HES signaling than fully active (A) cells while partially inactive (pI) cells exhibit more active VEGFR2* signaling than their fully inhibited counterparts. Thus simulations show that monitoring endogenous DLL is not sufficient to distinguish these partial states at the angiogenic front; it is critical to complement this measurement with at least one key readout of notch activity. It is interesting to note that although there are gradients in the expression patterns of the signaling proteins *within* the four main categories, the transition *between* categories is abrupt and in accordance with the bistable switches observed between these transitions.

## Discussion

In this paper, we use computational modeling to study the dynamics of EC patterning at a local cellular level. We explore several different well controlled scenarios to arrive at a set of novel predictions: (a) EC patterning speed is highly variable along the sprouting front depending on environmental/umwelt conditions of the cells in question, (b) EC patterning is made flexible and efficient due to the presence of multiple bistable transitions and partial states and (c) the timing and presence of the partial states is regulated by both external and internal cellular factors that modulate notch-DLL signaling.

Interestingly, our simulations revealed the presence of stable partial states (pI-partial inactive, pA-partial active) that are sensitive to external (neighboring DLL) and internal (Sirt1/Lfng) modulators. Presence of a multi-step switch allows the EC to react quickly and adapt sensitively to changing environmental conditions, and explains the quick patterning speeds observed by [7]. For example, a cell that starts as active could toggle between A↔pI at a lower threshold of lateral inhibition from its neighbors and then transition from pI↔I when the lateral inhibition still further increases (Fig 4). Due to the presence of bistability if for some reason the lateral inhibition values change, the cell can make the reversal switch i.e. pI↔A instead of making a complete A↔I transition. To our knowledge this is the first paper that has linked the presence of multiple bistability in EC patterning to EC overtaking and patterning seen in vivo. These predictions now also explain how some cells decide to overtake but then later do not commit completely and fall back to original position[7].

There are important biological ramifications of these *in silico* predictions that warrant further experimental studies: 1) Morphogenesis of the vasculature could create vastly different structures if cell state decisions were to take longer or move through partial states in local regions of the network. Indeed, our results are consistent with the hyper/hypo sprouting phenotypes observed when Sirt1/Lfng are perturbed *in vivo*. 2) Both R2 and notch (not DLL alone) are critical modulators of EC phenotypic outcomes, above and beyond patterning and migration. They also control proliferation, permeability, contact inhibition and inflammatory responses. Therefore, the previously unrecognized partial states warrant closer experimental inspection, as the activity of R2 and notch in these partial states could also lead to angiogenic context-dependent changes in this broader set of biological responses. 3) The model supports our recent claim that temporal modulation could offer a novel therapeutic strategy in diseases where vascular morphogenesis has become abnormal [8] and provides an extendable model with which to investigate this further in the future. Hypothetically, one could fine tune the tip/stalk balance and drive desired changes in branching patterns by targeting specific temporal modulators.

The presence of positive and negative feedback loops, coupled interactions (due to the lateral inhibition) and co-operativity in gene expression in our detailed 2-cell ODE model, make for highly non-linear behavior. Therefore, the system exhibits interesting multi-stable dynamics. Presence of bistability in notch-DLL signaling with respect to EC patterning has recently been shown by us [32], and others [24], [17]. Recently EC patterning has been shown to exhibit diseased ‘hybrid states’ [17] that result due to overexpression of Jagged-1, (another Notch ligand). In comparison using a detailed and well-documented VEGF-Notch-DLL4 signaling network we have shown in the current paper that EC patterning dynamics is more complicated and the presence of partial states does not solely depend only on the presence of Jagged. Our simulations based on physiologically relevant cellular umwelts and scenarios (e.g. VEGF gradient, neighbor cell movement etc.) indicate that the presence of partial states is an intrinsic feature of EC patterning. Partial states give the EC flexibility to make patterning decisions depending on their environment and also control the time taken to establish a pattern.

We find that external (neighboring DLL) and internal modulators (Sirt1 and Lfng) work in tandem to modulate the time spent in the partial states. Neighboring cell’s lateral inhibition coupled with internal Sirt1 (and Lfng) levels therefore work together to quickly adapt to the environment and dynamically change the EC patterning as seen in the cells of an angiogenic sprout. It is tempting to speculate that cells dynamically regulate their Sirt1/Lfng expression over time in order to be more adaptive, to avoid falling into the pI state for too long during position overtaking/cell rearrangement. Though outside of the scope of this study, the adaptive capacity of temporal modulator regulation would be interesting to explore in future studies.

It is to be noted that the partial states (pI and pA) are very close to the Inactive and Active states, respectively, in terms of their DLL concentration *alone*. However measuring all the species, especially proteins affected by the notch signaling (e.g. NICD and HE), reveal that a small jump in DLL concentrations at the partial states is coupled to a larger change in notch signaling proteins (Fig 7). Our simulations thus reveal that for a complete experimental characterization of the partial state, it is essential that DLL levels be measured together with nicd/HE.

Although in the current paper we have focused our analysis on the effects of three important modulators of notch-DLL signaling, the complexity of the angiogenic signaling system as a whole suggests that there may be a plethora of potential temporal modulating factors that fine tune cellular decision speeds and subsequently, the branching process itself. Therefore, we believe that these first, simple simulations detailed here will pave the way for many interesting studies into the temporal dimension and cross-talking factors altering cell dynamics during angiogenesis in the future.

## Supporting Information

S1 Fig Effect of static D_ext_ values on EC patterning states. The pattering states of the 2-cell model observed with changing D_ext1_ and D_ext2_ of neighboring cells. This result maps the EC patterning state, while its corresponding Fig 3D maps the EC pattering speed. Each cell in the matrix represents the difference in DLL levels of both cells i.e. DLL_cell1_-DLL_cell2_. When DLL_cell1_> DLL_cell2_, the 2-cell model patterns in A:I state (orange regions), when DLL_cell1_< DLL_cell2_ the 2-cell model patterns into I:A state (green regions). When both DLL_cell1_ & DLL_cell2_ are very high the 2-cell model patterns into I:I state (white). Apart from A and I, there are also partial states indicated by ∂ and shown as gradations in green and yellow. These represent the pI (partial inactive) and pA (partial active) states.
(EPS)

S2 Fig Effect of Lfng on EC patterning speeds. **(A)** EC patterning speed with different Lfng concentration. Lfng = 1c.u. optimal model, * and ** represent the threshold Lfng levels to transit from A:A →I:A/A:I and I:A/I:I→ A:A states respectively. Time series change in internal ΔDLL levels of the 2-cells representing state change at **(C)** low Lfng (Lfng = 0.5 c.u.) and **(D)** high Lfng (Lfng = 2 c.u.) values.
(EPS)

S3 Fig EC dynamics with perturbation of Sirt1 and Lfng. Bifurcation diagram of 2-cell model plotting **(A)** DLL_cell1_ and **(B)** DLL_cell2_ with Sirt1 as bifurcation parameter. Solid lines indicate a stable steady state and different colors represent each simulation done with a different Lfng value as indicated, while red dashed lines indicate unstable steady states.
(EPS)

S4 Fig Robustness analysis. Plotting the results of % bistable parameter sets when optimal parameter set is modified randomly between ±10% to ±50% from the nominal values. (See Materials and Methods for details).
(EPS)
